# De novo prediction of the genomic components and capabilities for microbial plant biomass degradation from (meta-)genomes

**DOI:** 10.1186/1754-6834-6-24

**Published:** 2013-02-15

**Authors:** Aaron Weimann, Yulia Trukhina, Phillip B Pope, Sebastian GA Konietzny, Alice C McHardy

**Affiliations:** 1 Max-Planck Research Group for Computational Genomics and Epidemiology, Max-Planck Institute for Informatics, University Campus E1 4, Saarbrücken, 66123, Germany; 2 Department of Chemistry, Biotechnology and Food Science, Norwegian University of Life Sciences, Post Office Box 5003, Ås, 1432, Norway; 3 Department of Algorithmic Bioinformatics, Heinrich Heine University Düsseldorf, Düsseldorf, 40225, Germany

## Abstract

**Background:**

Understanding the biological mechanisms used by microorganisms for plant biomass degradation is of considerable biotechnological interest. Despite of the growing number of sequenced (meta)genomes of plant biomass-degrading microbes, there is currently no technique for the systematic determination of the genomic components of this process from these data.

**Results:**

We describe a computational method for the discovery of the protein domains and CAZy families involved in microbial plant biomass degradation. Our method furthermore accurately predicts the capability to degrade plant biomass for microbial species from their genome sequences. Application to a large, manually curated data set of microbial degraders and non-degraders identified gene families of enzymes known by physiological and biochemical tests to be implicated in cellulose degradation, such as GH5 and GH6. Additionally, genes of enzymes that degrade other plant polysaccharides, such as hemicellulose, pectins and oligosaccharides, were found, as well as gene families which have not previously been related to the process. For draft genomes reconstructed from a cow rumen metagenome our method predicted Bacteroidetes-affiliated species and a relative to a known plant biomass degrader to be plant biomass degraders. This was supported by the presence of genes encoding enzymatically active glycoside hydrolases in these genomes.

**Conclusions:**

Our results show the potential of the method for generating novel insights into microbial plant biomass degradation from (meta-)genome data, where there is an increasing production of genome assemblages for uncultured microbes.

## Background

Lignocellulosic biomass is the primary component of all plants and one of the most abundant organic compounds on earth. It is a renewable, geographically distributed and a source of sugars, which can subsequently be converted into biofuels with low greenhouse gas emissions, such as ethanol. Chemically, it primarily consists of cellulose, hemicellulose and lignin. Saccharification - the process of degrading lignocellulose into the individual component sugars - is of considerable biotechnological interest. Several mechanical and chemical procedures for saccharification have been established; however, all are relatively expensive, slow and inefficient [1]. An alternative approach is realized in nature by various microorganisms, which use enzyme-driven lignocellulose degradation to generate sugars as sources of carbon and energy. The search for novel enzymes allowing an efficient breakdown of plant biomass has therefore attracted considerable interest [2-5]. In particular, the discovery of novel cellulases for saccharification is considered crucial in this context [6]. However, the complexity of the underlying biological mechanisms and the lack of robust enzymes that can be economically produced in larger quantities currently still prevent industrial application.

For some lignocellulose-degrading species, carbohydrate-active enzymes (CAZymes) and protein domains implicated in lignocellulose degradation are well known. Many of these have been recognized by physiological and biochemical tests as being relevant for the biochemical process of cellulose degradation itself, such as the enzymes of the glycoside hydrolase (GH) families GH6 and GH9 and the endoglucanase-containing family GH5. Two well-studied paradigms are currently known for microbial cellulose degradation: The ‘free-enzyme system’ is realized in most aerobic microbes and entails secretion of a set of cellulases to the outside of the cell. In anaerobic microorganisms large multi-enzyme complexes, known as cellulosomes, are assembled on the cell surface and catalyze degradation. In both cases, the complete hydrolysis of cellulose requires endoglucanases (GH5 and GH9), which are believed to target non-crystalline regions, and exo-acting cellobiohydrolases, which attack crystalline structures from either the reducing (GH7 and GH48) or non-reducing (GH6) end of the beta-glucan chain. However, in the genomes of some plant biomass-degrading species, homologs of such enzymes have not been found. Recent genome analyses of the lignocellulose-degrading microorganisms, such as the aerobe *Cytophaga hutchinsonii*[7], the anaerobe *Fibrobacter succinogenes*[8,9] and the extreme thermophile anaerobe *Dictyoglomus turgidum*[10] have revealed only GH5 and GH9 endoglucanases. Genes encoding exo-acting cellobiohydrolases (GH6 and GH48) and cellulosome structures (dockerins and cohesins) are absent.

Metagenomics offers the possibility of studying the genetic material of difficult-to-culture (i.e. uncultured) species within microbial communities with the capability to degrade plant biomass. Recent metagenome studies of the gut microbiomes of the wood-degrading higher termites (*Nasutitermes*), the Australian Tammar wallaby (*Macropus eugenii*) [11,12] and two studies of the cow rumen metagenome [13,14] have revealed new insights into the mechanisms of cellulose degradation in uncultured organisms and microbial communities. Microbial communities of different herbivores have been shown to be dominated by lineages affiliated to the Bacteroidetes and Firmicutes, of which different Bacteroidetes lineages exhibited endoglucanse activity [11,15]. Notably, exo-acting families and cellulosomal structures have a low representation or are entirely absent from gut metagenomes sequenced to date. Thus, current knowledge about genes and pathways involved in plant biomass degradation in different species, particularly uncultured microbial ones, is still incomplete.

We describe a method for the *de novo* discovery of protein domains and CAZy families associated with microbial plant biomass degradation from genome and metagenome sequences. It uses protein domain and gene family annotations as input and identifies those domains or gene families, which in concert are most distinctive for the lignocellulose degraders. Among the gene and protein domains identified with our method were known key genes of plant biomass degradation. Additionally, it identified several novel protein domains and gene families as being relevant for the process. These might represent novel leads towards elucidating the mechanisms of plant biomass degradation for the currently less well understood microbial species. Our method furthermore can be used to identify plant biomass-degrading species from the genomes of cultured or uncultured microbes. Application to draft genomes assembled from the metagenome of a switchgrass-adherent microbial community in cow rumen predicted genomes from several Bacteroidales lineages which encode active glycoside hydrolases and a relative to a known plant biomass degrader to represent lignocellulose degraders.

In technical terms, our method selects the most informative features from an ensemble of L1-regularized L2-loss linear Support Vector Machine (SVM) classifiers, trained to distinguish genomes of cellulose-degrading species from non-degrading species based on protein family content. Protein domain annotations are available in public databases and new protein sequences can be rapidly annotated with Hidden Markov Models (HMMs) or - somewhat slower - with BLAST searches of one protein versus the NCBI-nr database [16]. Co-occurrence of protein families in the biomass-degrading fraction of samples and an absence of these families within the non-degrading fraction allows the classifier to link these proteins to biomass degradation *without* requiring sequence homology to known proteins involved in lignocellulose degradation. Classification with SVMs has been previously used successfully for phenotype prediction from genetic variations in genomic data. In Beerenwinkel *et al.*[17], support vector regression models were used for predicting phenotypic drug resistance from genotypes. SVM classification was used by Yosef *et al.*[18] for predicting plasma lipid levels in baboons based on single nucleotide polymorphism data. In Someya *et al.*[19], SVMs were used to predict carbohydrate-binding proteins from amino acid sequences. The SVM [20,21] is a discriminative learning method that infers, in a supervised fashion, the relationship between input features (such as the distribution of conserved gene clusters or single nucleotide polymorphisms across a set of sequence samples) and a target variable, such as a certain phenotype, from labeled training data. The inferred function is subsequently used to predict the value of this target variable for new data points. This type of method makes no *a priori* assumptions about the problem domain. SVMs can be applied to datasets with millions of input features and have good generalization abilities, in that models inferred from small amounts of training data show good predictive accuracy on novel data. The use of models that include an L1-regularization term favors solutions in which few features are required for accurate prediction. There are several reasons why sparseness is desirable: the high dimensionality of many real datasets results in great challenges for processing. Many features in these datasets are usually non-informative or noisy, and a sparse classifier can lead to a faster prediction. In some applications, like ours, a small set of relevant features is desirable because it allows direct interpretation of the results.

## Results

**Figure 1:**
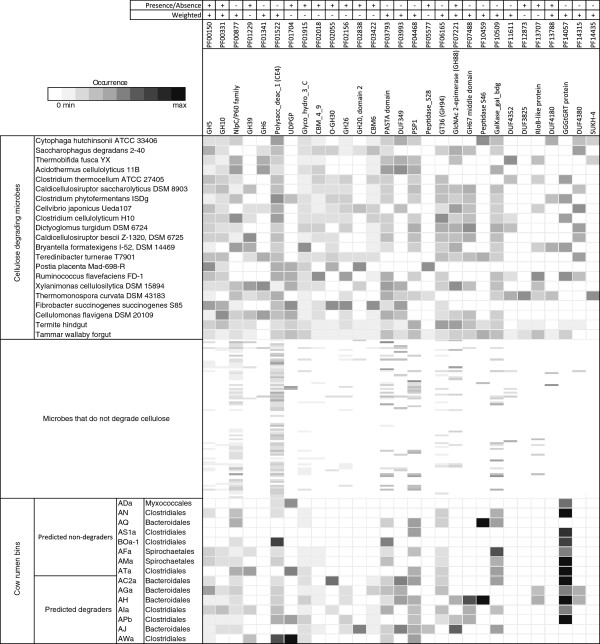
**Frequencies of the selected Pfam families in the individual genomes and metagenomes.** The data for each entry are rescaled by the total number of Pfam domains annotated to the microbial genome or metagenome. The color scale from grey to black indicates domain families that are present in low to high amounts, respectively. White indicates absent protein domains. The signs “+” and “-” indicate whether a protein domain was chosen in the respective experiment.

**Figure 2:**
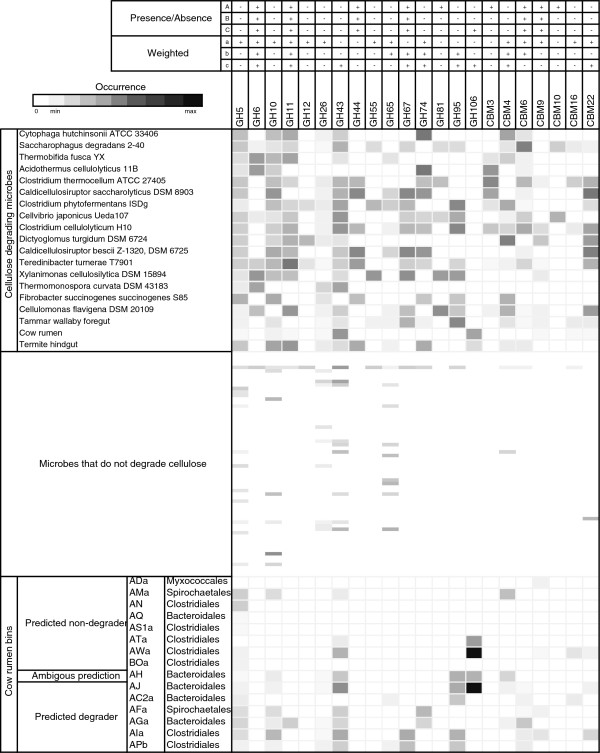
**Frequencies of selected glycoside hydrolase (GH) families and carbohydrate binding modules (CBMs) in the (meta-) genome sequences.** The data for each entry are rescaled by the total number of GH and CBM domains annotated to the microbial genome or metagenome. The coloring from black to grey indicates domains that are present in high to low amounts, respectively. White indicates absent domain families (“A”, “a”, “B”, “b”, “C”, “c” as described in Table 1).

We trained an ensemble of SVM classifiers to distinguish between plant biomass-degrading and non-degrading microorganisms based on either Pfam domain or CAZY gene family annotations (see Methods section for the training and evaluation of the SVM classification ensemble). We used a manually curated data set of 104 microbial (meta-)genome sequence samples for this purpose, which included 19 genomes and 3 metagenomes of lignocellulose degraders and 82 genomes of non-degraders (Figure 1, Figure 2, Additional file 1: Table S1). Fungi are known to use several enzymes for plant biomass degradation for which the corresponding genes are not found in prokaryotic genomes and vice versa, while other genes are shared by prokaryotic and eukaryotic degraders. To investigate similarities and differences detectable with our method, we included the genome of lignocellulose degrading fungus *Postia placenta* into our analysis. After training, we identified the most distinctive protein domains and CAZy families of plant biomass degraders from the resulting models. We compared these protein domains and gene families with known plant biomass degradation genes. We furthermore applied our method to identify plant biomass degraders among 15 draft genomes from the metagenome of a microbial community adherent to switch grass in cow rumen.

### Distinctive Pfam domains of microbial plant biomass degraders

For the training of a classifier which distinguishes between plant biomass-degrading and non-degrading microorganisms we used Pfam annotations of 101 microbial genomes and two metagenomes. This included metagenomes of microbial communities from the gut of a wood-degrading higher termite and from the foregut of the Australian Tammar Wallaby as examples for plant biomass-degrading communities. Furthermore, 19 genomes of microbial lignocellulose degraders were included - of the phyla Firmicutes (7 isolate genome sequences), Actinobacteria (5), Proteobacteria (3), Bacteroidetes (1), Fibrobacteres (1), Dictyoglomi (1) and Basidiomycota (1). Eighty-two microbial genomes annotated to not possess the capability to degrade lignocellulose were used as examples of non-lignocellulose-degrading microbial species (Additional file 1: Table S1).

We assessed the value of information about the presence or absence of protein domains for distinguishing lignocellulose degraders from non-degraders. With the respective classifier, eSVM_bPFAM_, each microbial (meta-)genome sequence was represented by a feature vector with the features indicating the presence or absence of Pfam domains (see Methods). The nested cross-validation macro-accuracy of eSVM_bPFAM_ in distinguishing plant biomass-degrading from non-degrading microorganisms was 0.91. This corresponds to 94% (97 of 103) of the (meta-)genome sequences being classified correctly. Only three of the 21 cellulose-degrading samples and three of the non-degraders were misclassified (Table 1, Table 2). Among these were four Actinobacteria and one genome affiliated with the Basidiomycota and Theromotogae each.

**Table 1:** Misclassified species in the SVM analyses

	**eSVM**_**bPFAM**_	**eSVM**_**CAZY_B**_
*False negatives*	*Postia placenta Mad-698-R*	*Thermomonospora curvata DSM 43183*
	*Xylanimonas cellulosilytica DSM 15894*	
	*Thermomonospora curvata DSM 43183*	
*False positives*	*Actinosynnema mirum 101*	*Actinosynnema mirum 101*
	*Arthrobacter aurescens TC1*	
	*Thermotoga lettingae TMO*	

Shown are species which were misclassified with the eSVM_CAZY_B_ and the eSVM_bPFAM_ classifiers. Contrary to previous beliefs [22], recent literature indicates in agreement with our predictions that *T. curvata* is a non-degrader. Furthermore, recent evidence supports that *A. mirum* is a lignocellulose degrader, which has not been previously described [23].

We identified the Pfam domains with the greatest importance for assignment to the lignocellulose-degrading class by eSVM_bPFAM_ (Figure 1; see Methods for the feature selection algorithm). Among these are several protein domains known to be relevant for plant biomass degradation. One of them is the GH5 family, which is present in all of the plant biomass-degrading samples. Almost all activities determined within this family are relevant to plant biomass degradation. Because of its functional diversity, a subfamily classification of the GH5 family was recently proposed [24]. The carbohydrate-binding modules CBM_6 and CBM_4_9 were also selected. Both families are Type B carbohydrate-binding modules (CBMs), which exhibit a wide range of specificities, recognizing single glycan chains comprising hemicellulose (xylans, mannans, galactans and glucans of mixed linkages) and/or non-crystalline cellulose [25]. Type A CBMs (e.g. CBM2 and CBM3), which are more commonly associated with binding to insoluble, highly crystalline cellulose, were not identified as relevant by eSVM_bPFAM_. Furthermore, numerous enzymes that degrade non-cellulosic plant structural polysaccharides were identified, including those that attack the backbone and side chains of hemicellulosic polysaccharides. Examples include the GH10 xylanases and GH26 mannanases. Additionally, enzymes that generally display specificity for oligosaccharides were selected, including GH39 β-xylosidases and GH3 enzymes.

We subsequently trained a classifier - eSVM_fPFAM_ - with a weighted representation of Pfam domain frequencies for the same data set. The macro-accuracy of eSVM_fPFAM_ was 0.84 (Table 2); lower than that of the eSVM_bPFAM_; with nine misclassified samples (4 Actinobacteria, 2 Bacteroidetes, 1 Basidiomycota, 1 Thermotogae phyla and the Tammar Wallaby metagenome). Again, we determined the most relevant protein domains for identifying a plant biomass-degrading sequence sample from the models by feature selection. Among the most important protein families were, as before, GH5, GH10 and GH88 (PF07221: N-acylglucosamine 2-epimerase) (Figure 1). GH6, GH67 and CE4 acetyl xylan esterases (“accessory enzymes” that contribute towards complete hydrolysis of xylan) were only relevant for prediction with the eSVM_fPFAM_ classifier. Additionally, both models specified protein domains not commonly associated with plant biomass degradation as being relevant for assignment, such as the lipoproteins DUF4352 and PF00877 (NlpC/P60 family) and binding domains PF10509 (galactose-binding signature domain) and PF03793 (PASTA domain) (Figure 1).

### Distinctive CAZy families of microbial plant biomass degraders

We searched for distinctive CAZy families of microbial plant biomass degraders with our method. CAZy families include glycoside hydrolases (GH), carbohydrate-binding modules (CBM), glycosyltransferases (GT), polysaccharide lyases (PL) and carbohydrate esterases (CE). The annotations from the CAZy database comprised 64 genomes of non-lignocellulose-degrading species and 16 genomes of lignocellulose-degraders. There were no CAZy annotations available for the remaining genomes. In addition, we included the metagenomes of the gut microbiomes of the Tammar wallaby (TW), the wood-degrading higher termite and of the cow rumen microbiome (Additional file 1: Table S1). We evaluated the value of information about the presence or absence of CAZy domains, or of their relative frequencies for identification of lignocellulose-degrading microbial (meta-)genomes in the following experiments:

1) By training of the classifiers eSVM_CAZY_A_ (presence/absence) and eSVM_CAZY_a_ (counts), based on genome annotations with all CAZy families.

2) By training of the classifiers eSVM_CAZY_B_ (presence/absence) and eSVM_CAZY_b_ (counts), based on the annotations of the genomes and the TW sample with all CAZy families, except for the GT family members, which were not annotated for the TW sample.

3) By training of the classifiers eSVM_CAZY_C_ (presence/absence) and eSVM_CAZY_c_ (counts) with the entire data set based on GH family and CBM annotations, as these were the only ones available for the three metagenomes.

The macro-accuracy of these classifiers ranged from 0.87 to 0.96, similar to the Pfam-domain-based models (Table 2). Notably, almost exclusively Actinobacteria were misclassified by the eSVM_CAZY_ classifiers, except for the Firmicute *Caldicellulosiruptor saccharolyticus*. The best classification results were obtained with the presence-absence information for all CAZy families except for the GT families of the microbial genomes and the TW sample. In this setting (eSVM_CAZY_B_) only two species (*Thermomonospora curvata* and *Actinosynnema mirum*) were misclassified (Table 1). These species remained misclassified with all six classifiers.

**Table 2:** Accuracy of classifying microbes as lignocellulose-degraders or non-degraders

	**Presence/absence of Pfam domains**	**Weighted Pfam domain representation**	**Presence/absence CAZy family representation**			**Weighted CAZy family representation**		
			**A**	**B**	**C**	**a**	**b**	**c**
**nCV macro-accuracy**	0.91	0.84	0.90	0.96	0.94	0.91	0.93	0.87
**nCV recall**	0.86	0.73	0.81	0.94	0.90	0.88	0.88	0.79
**nCV true negative rate**	0.96	0.96	0.98	0.98	0.98	0.95	0.98	0.95

L1-regularized SVMs were trained with Pfam domain or CAZY family (meta-)genome annotations. Capital letters denote classifiers trained based on the presence or absence of CAZy families and small letters indicate classifiers trained based on the relative abundances of CAZy families in annotations. Abbreviations “A”, “a”,” B”, “b”, “C”, “c” denote the following: Classifiers “A“,“a“ were trained with annotations of all CAZy families for 16 microbial genomes; Classifiers “B“,“b“ were trained with annotations for all CAZy families, except for the GT family members (which were not annotated for the Tammar Wallaby metagenome), for 16 genomes and the TW metagenome of plant biomass degraders; Classifiers “C“,“c“ were trained with annotations for the GH families and CBMs for the 16 microbial genomes and three metagenomes of plant biomass degraders, as only these were annotated for the metagenomes. All CAZy-based classifiers were trained with available annotations for 64 genomes of non-biomass degraders. The Pfam-based classifiers were trained with 21 (meta-)genomes of biomass-degraders and 82 microbial genomes of non-degraders. For more details on the experimental set-up and the evaluation measures shown see the Methods section on performance evaluation.

Using feature selection, we determined the CAZy families from the six eSVM_CAZy_ classifiers that are most relevant for identifying microbial cellulose-degraders. Many of these GH families and CBMs are present in all (meta-)genomes (Figure 2). This analysis identified further gene families known to be relevant for plant biomass degradation. Among them are cellulase-containing families (GH5, GH6, GH12, GH44, GH74), hemicellulase-containing families (GH10, GH11, GH26, GH55, GH81, GH115), families with known oligosaccharide/side-chain-degrading activities (GH43, GH65, GH67, GH95) and several CBMs (CBM3, -4, -6, -9, -10, -16, -22, -56). Several of these (GH6, GH11, GH44, GH67, GH74, CBM4, CBM6, CBM9) were consistently identified by at least half of the six classifiers as distinctive for plant biomass degraders. These might be considered signature genes of the plant biomass-degrading microorganisms we analyzed. Additionally, several GT, PL and CE domains were identified as relevant (eSVM_CAZY_A_: PL1, PL11 and CE5, “eSVM_CAZY_B_: CE5; eSVM_CAZY_a_: GT39, PL1 and CE2, eSVM_CAZY_b_: none). These CAZy families, as well as GH115 and CBM56, are not included in Figure 2, as they are not annotated for all sequences.

### Identification of plant biomass degraders from a cow rumen metagenome

We used our method to predict the plant biomass-degrading capabilities for 15 draft genomes of uncultured microbes reconstructed from the metagenome of a microbial community adherent to switchgrass in cow rumen [14] (see Methods for the classification with an ensemble of SVM classifiers). The draft genomes represent genomes with more than 50% of the sequence reconstructed by taxonomic binning of the metagenome sample. The microbial community adherent to switchgrass is likely to be enriched with plant biomass degraders, as it was found to differ from the rumen fluid community in its taxonomic composition and degradation of switch grass after incubation in cow rumen had occurred. For identification of plant biomass-degrading microbes, we classified each draft genome individually with the eSVM_bPFAM_ and eSVM_CAZY_B_ models, which had the highest macro-accuracy based on Pfam domain or CAZy family annotations, respectively. The eSVM_bPFAM_ classifier assigned seven of the draft genomes to plant biomass degraders (Table 3). One of these, genome *APb*, was found by 16S rRNA analysis to be related to the fibrolytic species *Butyrivibrio fibrisolvens.* Four others (*AC2a*, *AGa*, *AJ* and *AH*) are of the order of Bacteroidales, and include all but one draft genomes affiliated to the Bacteroidales. The 6^th^ and 7^th^ predicted degrader, represented by genome *AIa* and *AWa*, belong to the Clostridiales, like genome *APb*. The eSVM_CAZY_B_ classifier also assigned five of these genomes to the plant biomass degraders. Additionally it classified genome *AH* as plant biomass-degrading, while being ambiguous in the assignment of *AFa* (Table 3). To validate these predictions, we searched the draft genomes for genes encoding 51 enzymatically active glycoside hydrolases characterized from the same rumen dataset (for the results of these experiments see Figure three in Hess *et al.*[14]). Genomes *AGa*, *AC2a*, *AJ* and *AIa* were all linked to different enzymes of varying specificities (Table 3). *AC2a* was linked to cellulose degradation, specifically to a carboxymethyl cellulose (CMC)-degrading GH5 endoglucanase as well as GH9 enzyme capable of degrading insoluble cellulosic substrates such as Avicel®. *AIa* demonstrated capabilities towards xylan and soluble cellulosic substrates with affiliations to four GH10 xylanases. Both *AGa* and *AJ* demonstrated broader substrate versatility and were linked to enzymes with capabilities towards cellulosic substrates CMC and Avicel® (GH5, GH9 and GH26), hemicellulosic substrates lichenan (β-1,3, β-1,4 β-glucan) and xylan (GH5, GH9 and GH10), as well as the natural feedstocks miscanthus and switchgrass (GH5 and GH9). Importantly, no carbohydrate-active enzymes were affiliated to draft genomes that were predicted to not possess plant biomass-degrading capabilities (Table 3). Overall, assignments were largely consistent between the two classifiers and supporting evidence for the capability to degrade plant biomass was found for five of the predicted degraders.

**Table 3:** Prediction of the plant biomass degradation capabilities for 15 draft genomes

	**AC2a**	**AGa**	**AIa-2**	**AJ**	**APb**	**AFa**	**AH**	**AWa**	**ADa**	**AMa**	**AN**	**AQ**	**AS1**	**ATa**	**BOa**
eSVM_CAZY_B_	++	++	++	+	++	++	0	- -	- -	- -	- -	- -	- -	- -	- -
eSVM_bPFAM_	++	++	++	++	++	-	++	+	- -	-	- -	- -	- -	-	- -
CMC	GH5 (TW-33)	GH5 (TW-40)	GH10 (TW-34)	GH5 (TW-39)											
				GH26 (TW-10)											
				GH10 (TW-8)											
		GH5 (MH-2)													
XYL		GH10 (TW-25)	GH10 (TW-30)	GH10 (TW-8)											
			GH10 (TW-31)												
			GH10 (TW-37)												
SWG		GH5 (TW-40)													
		GH5 (MH-2)													
MIS	GH9 (TW-64)	GH5 (TW-40)		GH5 (TW-39)											
		GH5 (MH-2)													
		GH9 (TW-50)													
AVI	GH9 (TW-64)	GH5 (TW-40)		GH5 (TW-39)											
		GH5 (MH-2)													
		GH9 (TW-50)													
LIC		GH5 (TW-40)		GH5 (TW-39)											
		GH5 (MH-2)													
		GH9 (TW-50)													

Genome reconstructions from the metagenome of a microbial community adherent to switchgrass in the cow rumen were obtained by taxonomic binning of assembled sequences in the original study. Symbols depict the prediction outcome of a voting committee of the 5 eSVM_CAZY_B_ and the eSVM_bPFAM_ classifiers with the best macro-accuracy (see text for the description of the classifiers). ++: genome classified as plant biomass degrader by all classifiers; +: genome classified as plant biomass degrader by 4 out of 5 classifiers; 0: ambiguous prediction; -: genome classified as not plant biomass degrader by 4 out of 5 classifiers; --: genome classified as not plant biomass degrader by all classifiers. For every draft genome, the presence of genes encoding glycoside hydrolases with verified enzymatic activity for different substrates in this study [14] is indicated. The genome and substrate names correspond to those of Figure 3 and Table S6 of the study.

Hydrolytic activity detected on:

(CMC) 1% (w/v) carboxymethyl cellulose agar.

(XYL) 1% (w/v) Xylan.

(SWG) 1% (w/v) IL-Switchgrass.

(MIS) 1% (w/v) IL-Miscanthus.

(AVI) 1% (w/v) IL-Avicel.

### Timing experiments

Our method uses annotations with Pfam domains or CAZy families as input. Generating these by similarity-searches with profile HMMs rather than with BLAST provides a better scalability for next-generation sequencing data sets. HMM databases such as dbCAN contain a representation of entire protein families rather than of individual gene family members, which largely decreases the number of entries one has to compare against. For example, searching the ORFs of the *Fibrobacter succinogenes* genome [26] for similarities to CAZy families with the dbCAN HMM models took 23 seconds on an Intel® Xeon® 1.6 GHz CPU. In comparison, searching for similarities to CAZy families by BLASTing the same set of ORFs against all sequences with CAZy family annotation of the NCBI non-redundant protein database (downloaded from http://csbl.bmb.uga.edu/dbCAN/ on April 19th 2011) on the same machine required approximately 1 hour and 55 minutes, a difference of two orders of magnitude. Because of their better scalability and also because they are well-established for identifying protein domains or gene families [27-29], we recommend the use of HMM-based similarities and annotations as input to our method.

## Discussion

We investigated the value of information about the presence-or-absence of CAZy families and Pfam protein domains, as well as information about their relative abundances, for the identification of lignocellulose degraders. Classifiers trained with CAZy family or Pfam domain annotations allowed an accurate identification of plant biomass degraders and determined similar domains and CAZy families as being most distinctive. Many of these are recognized by physiological and biochemical tests as being relevant for the biochemical process of cellulose degradation itself, such as GH6, members of the GH5 family and to a lesser extent GH44 and GH74. In contrast to widely accepted paradigms for microbial cellulose degradation, recent genome analysis of cellulolytic bacteria has identified examples (i.e. *Fibrobacter*) where there is an absence of genes encoding exo-acting cellobiohydrolases (GH6 and GH48) and cellulosome structures [30]. In addition, these exo-acting families and cellulosomal structures have had a low representation or are entirely absent from sequenced gut metagenomes. Our method also finds the exo-acting cellobiohydrolases GH7 and GH48 to be less important. GH7 represents fungal enzymes, so its absence makes sense; however, the lower importance assigned to GH48 is interesting. The role of GH48 is believed to be of high importance, although recent research has raised questions. Olson *et al.*[31] have found that a complete solubilization of crystalline cellulose can occur in *Clostridium thermocellum* without the expression of GH48, albeit at significantly lower rates. Furthermore, genome analysis of cellulose-degrading microbes *Cellvibrio japonicus*[32] and *Saccharophagus degradans*[33] have determined the presence of only non-reducing end enzymes (GH6) and an absence of a reducing end cellobiohydrolase (GH48), suggesting that the latter are not essential for all cellulolytic enzyme systems.

While we have focused on cellulose degradation, our method has also identified enzymes that degrade other plant polysaccharides as being relevant, such as hemicellulose (GH10, GH11, GH12, GH26, GH55, GH81, CE4), pectins (PL1, GH88 and GH43), oligosaccharides (GH3, GH30, GH39, GH43, GH65, GH95) and the side-chains attached to noncellulosic polysaccharides (GH67, GH88, GH106). This was expected, since many cellulose-degrading microbes produce a repertoire of different glycoside hydrolases, lyases and esterases (see, for example, [32,33]) that target the numerous linkages that are present within different plant polysaccharides, which often exist in tight cross-linked forms within the plant cell wall. The results from our method add further weight to this. The observation of numerous CBMs being relevant in the CAZy analysis also agrees with previous findings that many different CBM-GH combinations are possible in bacteria. Moreover, recent reports have demonstrated that the targeting actions of CBMs have strong proximity effects within cell wall structures, i.e. CBMs directed to a cell wall polysaccharide (e.g. cellulose) other than the target substrate of their appended glycoside hydrolase (e.g. xylanase) can promote enzyme action against the target substrate (e.g. xylan) within the cell wall [34]. This provides explanations as to why cellulose-directed CBMs are appended to many non-cellulase cell wall hydrolases.

Several Pfam domains of unknown function (DUFs) or protein domains which have not previously been associated with cellulose degradation are predicted as being relevant. These include transferases (PF01704) and several putative lipoproteins (DUF4352), some of which have predicted binding properties (NlpC/P60 family: PF00877, PASTA domain: PF03793). The functions of these domains in relation to cellulose degradation are not known, but possibilities include binding to cellulose, binding to other components of the cellulolytic machinery or interaction with the cell surface.

Another result of our study are the classifiers for identifying microbial lignocellulose-degraders from genomes of cultured and uncultured microbial species reconstructed from metagenomes. Classification of draft genomes reconstructed from switchgrass-adherent microbes from cow rumen with the most accurate classifiers predicted six or seven of these to represent plant biomass-degrading microbes, including a close relative to the fibrolytic species *Butyrivibrio fibrisolvens*. Cross-referencing of all draft genomes against a catalogue of enzymatically active glycoside hydrolases provided a degree of method validation and was in majority agreement with our predictions. Four genomes (*AGa*, *AC2a*, *AJ* and *AIa*) predicted positive were linked to cellulolytic and/or hemicellulolytic enzymes, and importantly no genomes that were predicted negative were linked to carbohydrate-active enzymes from that catalogue of enzymatically active enzymes. Also, no connections to carbohydrate-active enzymes from that catalogue were observed for the three genomes (*AFa*,*AH* and *AWa*) where ambiguous predictions were made. As both draft genomes as well as the catalogue of carbohydrate active enzymes in cow rumen are incomplete, in addition to our training data not covering all plant-biomass-degrading taxa, such ambiguous assignments might be better resolvable with more information in the future.

We trained a previous version of our classifier with the genome of *Methanosarcina barkeri fusaro* incorrectly labeled as a plant biomass degrader, according to information provided by IMG. In cross-validation experiments, our method correctly assigned *M. barkeri* to be a non-plant biomass-degrading species. We labeled *Thermonospora curvata* as a plant biomass degrader and *Actinosynnema mirum* as non-degrader according to information from the literature (see Additional file 1: Table S1). Both were misassigned by all classifiers in the cross-validation experiments. However, in a recent work by Anderson *et al*. [23] it was shown that in cellulose activity assays *A. mirum* could degrade various cellulose substrates. In the same study, *T. curvata* did not show cellulolytic activity against any of these substrates, contrary to previous beliefs [22]. The authors found out that the cellulolytic *T. curvata* strain was in fact a *T. fusca* strain. Thus, our method could correctly assign both strains despite of the incorrect phenotypic labeling. The genome of *Postia placenta*, the only fungal plant biomass degrader of our data set was misassigned in the Pfam-based SVM analyses. Fungi possess cellulases not found in prokaryotic species [35] and might employ a different mechanism for plant biomass degradation [36,37]. Indeed, in our data set, *Postia placenta* is annotated with the cellulase-containing GH5 family and xylanase GH10, but the hemicellulase family GH26 does not occur. Furthermore, the (hemi-)cellulose binding CBM domains CBM6 and CBM_4_9, which were identified as being relevant for assignment to lignocellulose degraders with the eSVM_bPFAM_ classifier, are absent. All of the latter ones, GH26, CBM6 and especially CBM4 and CBM9, occur very rarely in eukaryotic genome annotations, according to the CAZy database.

## Conclusions

We have developed a computational technique for the identification of Pfam protein domains and CAZy families that are distinctive for microbial plant biomass degradation from (meta-)genome sequences and for predicting whether a (draft) genome of cultured or uncultured microorganisms encodes a plant biomass-degrading organism. Our method is based on feature selection from an ensemble of linear L1-regularized SVMs. It is sufficiently accurate to detect errors in phenotype assignments of microbial genomes. However, some microbial species remained misclassified in our analysis, which indicates that further distinctive genes and pathways for plant biomass degradation are currently poorly represented in the data and could therefore not be identified.

To identify a lignocellulose degrader from the currently available data, the presence of a few domains, many of which are already known, is sufficient. The identification of several protein domains which have so far not been associated with microbial plant biomass degradation in the Pfam-based SVM analyses as being relevant may warrant further scrutiny. A difficulty in our study was to generate a sufficiently large and correctly annotated dataset to reach reliable conclusions. This means that the results could probably be further improved in the future, as more sequences and information on plant biomass degraders become available. The method will probably also be suitable for identifying relevant gene and protein families of other phenotypes.

The prediction and subsequent validation of three Bacteroidales genomes to represent cellulose-degrading species demonstrates the value of our technique for the identification of plant biomass degraders from draft genomes from complex microbial communities, where there is an increasing production of genome assemblages for uncultured microbes. These to our knowledge represent the first cellulolytic Bacteroidetes-affiliated lineages described from herbivore gut environments. This finding has the potential to influence future cellulolytic activity investigations within rumen microbiomes, which has for the greater part been attributed to the metabolic capabilities of species affiliated to the bacterial phyla Firmicutes and Fibrobacteres.

## Methods

### Annotation

We annotated all protein coding sequences of microbial genomes and metagenomes with Pfam protein do-mains (Pfam-A 26.0) and Carbohydrate-Active Enzymes (CAZymes) [28,38]. The CAZy database contains information on families of structurally related catalytic modules and carbohydrate binding modules (CBMs) or (functional) domains of enzymes that degrade, modify or create glycosidic bonds. HMMs for the Pfam domains were downloaded from the Pfam database. Microbial and metagenomic protein sequences were retrieved from IMG 3.4 and IMG/M 3.3 [39,40]. HMMER 3 [41] with gathering thresholds was used to annotate the samples with Pfam domains. Each Pfam family has a manually defined gathering threshold for the bit score that was set in such a way that there were no false-positives detected. For annotation of protein sequences with CAZy families, the available annotations from the database were used. For annotations not available in the database, HMMs for the CAZy families were downloaded from dbCAN (http://csbl.bmb.uga.edu/dbcan) [42]. To be considered a valid annotation, matches to Pfam and dbCAN protein domain HMMs in the protein sequences were required to be supported by an e-value of at least 1e-02 and a bit score of at least 25. Additionally, we excluded matches to dbCAN HMMs with an alignment longer than 100 bp that did not exceed an e-value of 1e-04. Multiple matches of one and the same protein sequence against a single Pfam or dbCAN HMM exceeding the thresholds were counted as one annotation.

### Phenotype annotation of lignocellulose-degrading and non-degrading microbes

We defined genomes and metagenomes as originating from either lignocellulose-degrading or non-lignocellulose-degrading microbial species based on information provided by IMG/M and in the literature. For every microbial genome and metagenome, we downloaded the genome publication and further available articles (Additional file 1: Table S1). We did not consider genomes for which no publications were available. For cellulose-degrading species annotated in IMG, we verified these assignments based on these publications. We used text search to identify the keywords “cellulose”, “cellulase”, “carbon source”, “plant cell wall” or “polysaccharide” in the publications for non-cellulose-degrading species. We subsequently read all articles that contained these keywords in detail to classify the respective organism as either cellulose-degrading or non-degrading. Genomes that could not be unambiguously classified in this manner were excluded from our study.

### Classification with an ensemble of support vector machine classifiers

The SVM is a supervised learning method that can be used for data classification [20,21]. Here, we use an L1-regularized L2-loss SVM, which solves the following optimization problem for a set of instance-label pairs $\left({\vec{x}}_{i},y_{i}\right)$, ${\vec{x}}_{i}\in R^{n}$, *y*_*i*_ ∈ { -1, + 1}, *i* = 1, …, *l*:

(1) $$\min_{\vec{w}}||\vec{w}|{|}_{1}+C\sum_{i=1}^{l}{\left(\max\left(0,1-y_{i}{\vec{w}}^{T}{\vec{x}}_{i}\right)\right)}^{2},$$

where *C* ≥ 0 is a penalty parameter. This choice of the classifier and regularization term results in sparse models, where non-zero components of the weight vector $\vec{w}$ are important for discrimination between the classes [43]. SVM classification was performed using the LIBLINEAR package [44]. The components of ${\vec{x}}_{i}$ are either binary valued and represent the presence or absence of protein domains, or continuous-valued and represent the frequency of a particular protein domain or gene family relative to the total number of annotations. All features were normalized by dividing by the sum of all vector entries and subsequently scaled, such that the value of each feature was within the range [0,1]. The label +1 was assigned to genomes and metagenomes of plant biomass-degrading microorganisms, the label -1 to all sequences from non-degrading ones. Classification of the draft genomes assembled from the fiber-adherent microbial community from cow rumen was performed with a voting committee of multiple models with different settings for the penalty parameter *C* that performed comparably well. A majority vote of the 5 most accurate models was used here obtained in a single cross-validation run with different settings of the penalty parameter *C*.

### Performance evaluation

The assignment accuracy of a classifier was determined with a standard nested cross-validation (nCV) setup [45]. In nCV, an outer cross-validation loop is organized according to the leave-one-out principle: In each step, one data point is left out. In an inner loop, the optimal parameters for the model (here, the penalty parameter *C*) are sought, in a second cross-validation experiment with the remaining data points. For determination of the best setting for the penalty parameter *C*, values for *C* = 10^*x*^, *x* = -3.0, -2.5, -2.25, …, 0 were tried. Values of the parameter *C* larger than 1 were not tested extensively, as we found that they resulted in models with similar accuracies. This is in agreement with the Liblinear tutorial in the appendix of [44] which states that once the parameter C exceeds a certain value, the obtained models have a similar accuracy. The SVM with the penalty parameter setting yielding the best assignment accuracy was used to predict the class membership of the left out data point. The class membership predictions for all data points were used to determine the assignment accuracy of the classifier, based on their agreement with the correct assignments. For this purpose, the result of each leave-one-out experiment was classified as either a true positive (TP - correctly predicted lignocellulose degraders), true negative (TN - correctly predicted non-degraders), false positive (FP - non-degraders predicted to be degraders) or a false negative assignment (FN - degraders predicted to be non-degraders). The recall of the positive class and the true negative rate of the classifier were calculated according to the following equations:

(2) Recall=TPTP+FN

(3) $$\mathrm{True}\;\mathrm{negative}\;\mathrm{rate}=\frac{\mathrm{TN}}{\mathrm{TN}+\mathrm{FP}}$$

The average of the recall and the true negative rate, the macro-accuracy, was used as the assignment accuracy to assess the overall performance:

(4) MACC=Recall+Truenegativerate2

Subsequently, we identified the settings for the penalty parameter *C* with the best macro-accuracy by leave-one-out cross-validation. The parameter settings resulting in the most accurate models were used to each train a separate model on the entire data set. Prediction of the five best models were combined to form a voting committee and used for the classification of novel sequence samples such as the partial genome reconstructions from the cow rumen metagenome of switch-grass adherent microbes (see Additional file 2: Table S2 for an evaluation and meta-parameter settings of these ensembles of classifiers).

### Feature selection

An SVM model can be represented by a sparse weight vector $\vec{w}$. The positive and negative components of $\vec{w}$, the ‘feature weights’, specify the relative importance of the protein domains or CAZy families for discrimination between plant biomass-degrading and non-plant biomass-degrading microorganisms. To determine the most distinctive features for the positive class (that is, the lignocellulose degraders), we selected all features that received a positive weight in weight vectors of the majority of the five most accurate models. This ensemble of models was also used for classification of the cow rumen draft genomes of uncultured microbes (see Classification with a SVM).

## Competing interests

The authors declare that they have no competing interests.

## Authors’ contributions

AW, YT, PBP and ACM designed the study, interpreted the results and wrote the manuscript. AW and YT conducted the experiments under the supervision of ACM. SGAK and AW computed the CAZy family and protein domain annotations. All authors read and approved the final manuscript.

## Supplementary Material

Additional file 1: Table S1 Isolate strains and metagenome samples used in this study. The signs “+” and “-” indicate availability of CAZy or Pfam annotation data. The symbol * marks strains for which we provide another reference than the genome publication characterizing the metabolic capacities of the respective strain. Click here for file

Additional file 2: Table S2 Evaluation and meta-parameter settings of the ensembles of classifiers. The ensembles were used for feature selection and phenotype classification of the (draft) genomes and metagenomes. The macro-accuracy for each model for a discrete set of values for the parameter *C* was calculated in cross-validation experiments. The five best models were selected based on macro-accuracy. The mean of the exponentially transformed parameter *C* and the mean macro-accuracy for these five models are shown for all trained classifiers. For details on the different ensemble classifiers, see the Results section in the manuscript. Click here for file
