# Supplementary material for: De novo prediction of the genomic components and capabilities for microbial plant biomass degradation from (meta-)genomes
Source: Biotechnol Biofuels. 2013 Feb 15;6:24. doi: 10.1186/1754-6834-6-24 (PMC3585893; doi:10.1186/1754-6834-6-24)
Supplement: Additional file 1: Table S1 — Isolate strains and metagenome samples used in this study. The signs “+” and “-” indicate availability of CAZy or Pfam annotation data. The symbol * marks strains for which we provide another reference than the genome publication characterizing the metabolic capacities of the respective strain. [file 1754-6834-6-24-S1.doc]

**Supplementary Table S1. Isolate strains and metagenome samples used in this study.** The signs “+” and “-” indicate availability of CAZy or Pfam annotation data. The symbol * marks strains for which we provide another reference than the genome publication characterizing the metabolic capacities of the respective strain.

|  | | | | Pfam | CAZy | | | Reference |
| --- | --- | --- | --- | --- | --- | --- | --- | --- |
|  |  |  |  | Binary/  weighted | a | b | c |  |
| *Cellulose-degrading organisms* | *Meta-genomes* | 1 | *Macropus eugenii* gut microbiome (tammar wallaby) | + | – | + | + | Pope et al 2010) |
|  |  | 2 | Cow rumen microbiome | – | – | – | + | Brulc et al 2009 |
|  |  | 3 | Termite gut microbiome | + | – | – | + | Warnecke et al 2007 |
|  | *Genomes* | 1 | *Acidothermus cellulolyticus* 11B | + | + | | | Barabote et al 2009 |
|  |  | 2 | *Anaerocellum thermophilum* Z-1320, DSM 6725 | + | + | | | Kataeva et al 2009 |
|  |  | 3 | *Bryantella formatexigens* I-52, DSM 14469 | + | – | | | Wolin et al 2003 |
|  |  | 4 | *Caldicellulosiruptor saccharolyticus* DSM 8903 | + | + | | | Rainey et al 1994* |
|  |  | 5 | *Cellulomonas flavigena* 134, DSM 20109 | + | + | | | Abt et al 2010 |
|  |  | 6 | *Cellvibrio japonicus Ueda* 107 | + | + | | | DeBoy et al 2008 |
|  |  | 7 | *Clostridium cellulolyticum* H10 | + | + | | | Petitdemange et al 1984* |
|  |  | 8 | *Clostridium phytofermentans* ISDg | + | + | | | Warnick et al 2002* |
|  |  | 9 | *Clostridium thermocellum* ATCC 27405 | + | + | | | Feinberg et al 2011 |
|  |  | 10 | *Cytophaga hutchinsonii* ATCC 33406 | + | + | | | Xie et al 2007 |
|  |  | 11 | *Dictyoglomus turgidum* DSM 6724 | + | + | | | Brumm et al 2011 |
|  |  | 12 | *Fibrobacter succinogenes succinogenes* S85 | + | + | | | Bae et al 1993* |
|  |  | 13 | *Postia placenta* Mad-698-R | + | – | | | Martinez et al 2009 |
|  |  | 14 | *Ruminococcus flavefaciens* FD-1 | + | – | | | Berg Miller et al 2009 |
|  |  | 15 | *Saccharophagus degradans* 2-40 | + | + | | | Fraiberg et al 2010 |
|  |  | 16 | *Teredinibacter turnerae* T7901 | + | + | | | Yang et al 2009 |
|  |  | 17 | *Thermobifida fusca* YX | + | + | | | Lykidis et al 2007 |
|  |  | 18 | *Thermomonospora curvata* DSM 43183 | + | + | | | Chertkov et al 2011 |
|  |  | 19 | *Xylanimonas cellulosilytica* XIL07, DSM 15894 | + | + | | | Foster et al 2010 |
| *Non-cellulose degrading organisms* | *Genomes* | 1 | *Acetobacter pasteurianus* IFO 3283-01 | + | + | | | Azuma et al 2009 |
|  |  | 2 | *Acidimicrobium ferrooxidans* DSM 10331 | + | + | | | Clum et al 2009 |
|  |  | 3 | *Acidithiobacillus ferrooxidans* ATCC 23270 | + | + | | | Valdés et al 2008 |
|  |  | 4 | *Actinosynnema mirum* DSM 43827 | + | + | | | Land et al 2009 |
|  |  | 5 | *Agrobacterium tumefaciens* C58 (Cereon) | + | + | | | Wood et al 2001 |
|  |  | 6 | *Alcanivorax borkumensis* SK2 | + | + | | | Schneiker et al 2006 |
|  |  | 7 | *Alkalilimnicola ehrlichei* MLHE-1 | + | + | | | Hoeft et al 2007* * |
|  |  | 8 | *Alkaliphilus metalliredigens* QYMF | + | + | | | Fu et al 2009)* |
|  |  | 9 | *Archaeoglobus fulgidus* DSM 4304 | + | - | | | Klenk et al 1997 |
|  |  | 10 | *Arthrobacter aurescens* TC1 | + | + | | | Mongodin et al 2006 |
|  |  | 11 | *Azoarcus* sp. BH72 | + | + | | | Krause et al 2006 |
|  |  | 12 | *Azorhizobium caulinodans* ORS 571 | + | + | | | Liu et al 2011 |
|  |  | 13 | *Azotobacter vinelandii* DJ, ATCC BAA-1303 | + | + | | | Setubal et al 2009 |
|  |  | 14 | *Beijerinckia indica indica* ATCC 9039 | + | + | | | Tamas et al 2010 |
|  |  | 15 | *Candidatus amoebophilus asiaticus* 5a2 | + | + | | | Schmitz-Esser et al 2010 |
|  |  | 16 | *Chloroflexus aurantiacus* J-10-fl | + | + | | | Tang et al 2011 |
|  |  | 17 | *Chromobacterium violaceum* ATCC 12472 | + | + | | | Brazilian National Genome Project Consortium 2003 |
|  |  | 18 | *Comamonas testosteroni* KF-1 | + | - | | | Ma et al 2009 |
|  |  | 19 | *Cupriavidus taiwanensis* | + | - | | | Amadou et al 2008 |
|  |  | 20 | *Cyanothece* sp. ATCC 51142 | + | + | | | Welsh et al 2008 |
|  |  | 21 | *Dehalococcoides ethenogenes* 195 | + | + | | | Seshadri et al 2005 |
|  |  | 22 | *Desulfatibacillum alkenivorans* AK-01 | + | + | | | Callaghan et al 2012 |
|  |  | 23 | *Desulfitobacterium hafniense* DCB-2 | + | + | | | Shinoda et al 2006) |
|  |  | 24 | *Desulfohalobium retbaense* DSM 5692 | + | + | | | Spring et al 2010 |
|  |  | 25 | *Desulfomicrobium baculatum* DSM 4028 | + | + | | | Copeland et al 2009 |
|  |  | 26 | *Desulfotalea psychrophila* LSv54 | + | + | | | Rabus et al 2004 |
|  |  | 27 | *Desulfotomaculum reducens* MI-1 | + | + | | | Junier et al 2010 |
|  |  | 28 | *Diaphorobacter* sp. TPSYc | + | + | | | Byrne-Bailey et al 2010 |
|  |  | 29 | *Frankia alni* ACN14a | + | + | | | Normand et al 2007 |
|  |  | 30 | *Geobacter bemidjiensis* Bem | + | + | | | Aklujkar et al 2010 |
|  |  | 31 | *Hyperthermus butylicus* DSM 5456 | + | - | | | Brügger et al 2007 |
|  |  | 32 | *Klebsiella pneumoniae* 342 | + | + | | | Yi et al 2010 |
|  |  | 33 | *Lactobacillus salivarius salivarius* UCC118 | + | + | | | Jimenez et al 2010 |
|  |  | 34 | *Magnetococcus* sp. MC-1 | + | + | | | Schübbe et al 2009 |
|  |  | 35 | *Marinobacter aquaeolei* VT8 | + | + | | | Singer et al 2011* |
|  |  | 36 | *Mesorhizobium loti* MAFF303099 | + | + | | | Kaneko et al 2000 |
|  |  | 37 | *Metallosphaera sedula* DSM 5348 | + | - | | | Auernik et al 2008 |
|  |  | 38 | *Methanobrevibacter smithii* ATCC 35061 | + | - | | | Hansen et al 2011 |
|  |  | 39 | *Methanocaldococcus fervens* AG86 | + | - | | | Galperin and Cochrane 2009 |
|  |  | 40 | *Methanococcoides burtonii* DSM 6242 | + | - | | | Saunders et al 2003 |
|  |  | 41 | *Methanocorpusculum labreanum* Z | + | - | | | Anderson et al 2009 |
|  |  | 42 | *Methanoculleus marisnigri* JR1 | + | - | | | Anderson et al 2009 |
|  |  | 43 | *Methanopyrus kandleri* AV19 | + | - | | | Slesarev et al 2002 |
|  |  | 44 | *Methanosarcina acetivorans* C2A | + | - | | | Galagan et al 2002 |
|  |  | 45 | *Methanosphaera stadtmanae* DSM 3091 | + | - | | | Fricke et al 2006 |
|  |  | 46 | *Methylibium petroleiphilum* PM1 | + | + | | | Kane et al 2007 |
|  |  | 47 | *Methylocella silvestris* BL2 | + | + | | | Chen et al 2010 |
|  |  | 48 | *Nautilia profundicola* Am-H | + | + | | | Campbell et al 2009 |
|  |  | 49 | *Nitrobacter hamburgensis* X14 | + | + | | | Starkenburg et al 2008 |
|  |  | 50 | *Nitrosococcus oceani* ATCC 19707 | + | + | | | Klotz et al 2006 |
|  |  | 51 | *Nitrosomonas europaea* ATCC 19718 | + | + | | | Chain et al 2003 |
|  |  | 52 | *Nitrosopumilus maritimus* SCM1 | + | - | | | Walker et al 2010 |
|  |  | 53 | *Nitrosospira multiformis* ATCC 25196 | + | + | | | Norton et al 2008 |
|  |  | 54 | *Nostoc punctiforme* PCC 73102 | + | + | | | Meeks et al 2001 |
|  |  | 55 | *Paracoccus denitrificans* PD1222 | + | + | | | Siddavattam et al 2011 |
|  |  | 56 | *Parvibaculum lavamentivorans* DS-1 | + | + | | | Schleheck et al 2007 |
|  |  | 57 | *Pelotomaculum thermopropionicum* SI | + | + | | | Kosaka et al 2008 |
|  |  | 58 | *Persephonella marina* EX-H1 | + | + | | | Reysenbach et al 2009 |
|  |  | 59 | *Polaromonas naphthalenivorans* CJ2 | + | + | | | Yagi et al 2009 |
|  |  | 60 | *Pseudomonas mendocina* ymp | + | + | | | Guo et al 2011 |
|  |  | 61 | *Pyrobaculum aerophilum* IM2 | + | - | | | Fitz-Gibbon et al 2002 |
|  |  | 62 | *Pyrococcus abyssi* GE5 | + | - | | | (Cohen et al 2003 |
|  |  | 63 | *Rhizobium etli* CFN 42 | + | + | | | Fauvart et al 2011 |
|  |  | 64 | *Rhodobacter sphaeroides* KD131 | + | + | | | Porter et al 2011 |
|  |  | 65 | *Rhodococcus* sp. RHA1 | + | + | | | Takeda et al 2010 |
|  |  | 66 | *Rhodoferax ferrireducens* T118 | + | + | | | Risso et al 2009 |
|  |  | 67 | *Rhodospirillum rubrum* ATCC 11170 | + | + | | | Munk et al 2011 |
|  |  | 68 | *Sinorhizobium medicae* WSM419 | + | + | | | Reeve et al 2010 |
|  |  | 69 | *Slackia heliotrinireducens* DSM 20476 | + | + | | | Pukall et al 2009 |
|  |  | 70 | *Streptococcus thermophilus* LMD-9 | + | + | | | Sun et al 2011 |
|  |  | 71 | *Sulfolobus acidocaldarius* DSM 639 | + | - | | | Chen et al 2005 |
|  |  | 72 | *Sulfurospirillum deleyianum* DSM 6946 | + | + | | | Sikorski et al 2010 |
|  |  | 73 | *Synechococcus elongatus* PCC 7942 | + | + | | | Holtman et al 2005 |
|  |  | 74 | *Synechococcus* sp. CC9605 | + | + | | | Jenkins et al 2006)* |
|  |  | 75 | *Syntrophomonas wolfei wolfei* Goettingen | + | + | | | Sieber et al 2010 |
|  |  | 76 | *Syntrophus aciditrophicus* SB | + | + | | | McInerney et al 2007 |
|  |  | 77 | *Thermotoga lettingae* TMO | + | + | | | Zhaxybayeva et al 2009 |
|  |  | 78 | *Thioalkalivibrio* sp. HL-EbGR7 | + | + | | | Muyzer et al 2011 |
|  |  | 79 | *Thiobacillus denitrificans* ATCC 25259 | + | + | | | Beller et al 2006 |
|  |  | 80 | *Thiomicrospira crunogena* XCL-2 | + | + | | | Scott et al 2006 |
|  |  | 81 | *Thiomicrospira denitrificans* ATCC 33889 | + | + | | | Sievert et al 2008 |
|  |  | 82 | *Zymomonas mobilis mobilis* ZM4 | + | - | | | Pappas et al 2011 |

References

1. Pope PB, Denman SE, Jones M, Tringe SG, Barry K, Malfatti SA, McHardy AC, Cheng JF, Hugenholtz P, McSweeney CS, Morrison M: **Adaptation to herbivory by the Tammar wallaby includes bacterial and glycoside hydrolase profiles different from other herbivores.** *Proceedings of the National Academy of Sciences of the United States of America* 2010, **107:**14793-14798.

2. Brulc JM, Antonopoulos DA, Miller ME, Wilson MK, Yannarell AC, Dinsdale EA, Edwards RE, Frank ED, Emerson JB, Wacklin P, et al: **Gene-centric metagenomics of the fiber-adherent bovine rumen microbiome reveals forage specific glycoside hydrolases.** *Proceedings of the National Academy of Sciences of the United States of America* 2009, **106:**1948-1953.

3. Warnecke F, Luginbuhl P, Ivanova N, Ghassemian M, Richardson TH, Stege JT, Cayouette M, McHardy AC, Djordjevic G, Aboushadi N, et al: **Metagenomic and functional analysis of hindgut microbiota of a wood-feeding higher termite.** *Nature* 2007, **450:**560-565.

4. Barabote RD, Xie G, Leu DH, Normand P, Necsulea A, Adney WS, Xu XC, Lapidus A, Daubin V, Me C, et al: **Complete genome of the cellulolytic thermophile Acidothermus cellulolyticus 11B provides insights into its ecophysiological and evolutionary adaptations.** *Genome Research* 2009**:**1033-1043.

5. Kataeva Ia, Yang S-J, Dam P, Poole FL, Yin Y, Zhou F, Chou W-c, Xu Y, Goodwin L, Sims DR, et al: **Genome sequence of the anaerobic, thermophilic, and cellulolytic bacterium "Anaerocellum thermophilum" DSM 6725.** *Journal of bacteriology* 2009, **191:**3760-3761.

6. Wolin MJ, Miller TL, Collins MD, Lawson PA: **Formate-dependent growth and homoacetogenic fermentation by a bacterium from human feces: description of Bryantella formatexigens gen. nov., sp. nov.** *Applied and environmental microbiology* 2003, **69:**6321-6326.

7. Rainey FA, Donnison AM, Janssen PH, Saul D, Rodrigo A, Bergquist PL, Daniel RM, Stackebrandt E, Morgan HW: **Description of Caldicellulosiruptor saccharolyticus gen. nov., sp. nov: an obligately anaerobic, extremely thermophilic, cellulolytic bacterium.** *FEMS microbiology letters* 1994, **120:**263-266.

8. Abt B, Foster B, Lapidus A, Clum A, Sun H, Pukall Ru, Lucas S, Glavina Del Rio T, Nolan M, Tice H, et al: **Complete genome sequence of Cellulomonas flavigena type strain (134).** *Standards in genomic sciences* 2010, **3:**15-25.

9. DeBoy RT, Mongodin EF, Fouts DE, Tailford LE, Khouri H, Emerson JB, Mohamoud Y, Watkins K, Henrissat B, Gilbert HJ, Nelson KE: **Insights into plant cell wall degradation from the genome sequence of the soil bacterium Cellvibrio japonicus.** *Journal of bacteriology* 2008, **190:**5455-5463.

10. Petitdemange E, Biologique LDC, Ce V-l-n, Bacte C: **Clostridium cellulolyticum sp. nov. , a Cellulolytic, Mesophilic Species from Decayed Grass.** *International Journal* 1984**:**155-159.

11. Warnick TA, Methe BA, Leschine SB: **Clostridium phytofermentans sp. nov., a cellulolytic mesophile from forest soil.** *International journal of systematic and evolutionary microbiology* 2002, **52:**1155-1160.

12. Feinberg L, Foden J, Barrett T, Davenport KW, Bruce D, Detter C, Tapia R, Han C, Lapidus A, Lucas S, et al: **Complete genome sequence of the cellulolytic thermophile Clostridium thermocellum DSM1313.** *Journal of bacteriology* 2011, **193:**2906-2907.

13. Xie G, Bruce DC, Challacombe JF, Chertkov O, Detter JC, Gilna P, Han CS, Lucas S, Misra M, Myers GL, et al: **Genome sequence of the cellulolytic gliding bacterium Cytophaga hutchinsonii.** *Applied and environmental microbiology* 2007, **73:**3536-3546.

14. Brumm P, Hermanson S, Hochstein B, Boyum J, Hermersmann N, Gowda K, Mead D: **Mining Dictyoglomus turgidum for enzymatically active carbohydrases.** *Applied biochemistry and biotechnology* 2011, **163:**205-214.

15. Bae HD, McAllister Ta, Yanke J, Cheng KJ, Muir aD: **Effects of Condensed Tannins on Endoglucanase Activity and Filter Paper Digestion by Fibrobacter succinogenes S85.** *Applied and environmental microbiology* 1993, **59:**2132-2138.

16. Martinez D, Challacombe J, Morgenstern I, Hibbett D, Schmoll M, Kubicek CP, Ferreira P, Ruiz-Duenas FJ, Martinez AT, Kersten P, et al: **Genome, transcriptome, and secretome analysis of wood decay fungus Postia placenta supports unique mechanisms of lignocellulose conversion.** *Proceedings of the National Academy of Sciences of the United States of America* 2009, **106:**1954-1959.

17. Berg Miller ME, Antonopoulos Da, Rincon MT, Band M, Bari A, Akraiko T, Hernandez A, Thimmapuram J, Henrissat B, Coutinho PM, et al: **Diversity and strain specificity of plant cell wall degrading enzymes revealed by the draft genome of Ruminococcus flavefaciens FD-1.** *PloS one* 2009, **4:**e6650.

18. Fraiberg M, Borovok I, Weiner RM, Lamed R: **Discovery and characterization of cadherin domains in Saccharophagus degradans 2-40.** *Journal of bacteriology* 2010, **192:**1066-1074.

19. Yang JC, Madupu R, Durkin aS, Ekborg Na, Pedamallu CS, Hostetler JB, Radune D, Toms BS, Henrissat B, Coutinho PM, et al: **The complete genome of Teredinibacter turnerae T7901: an intracellular endosymbiont of marine wood-boring bivalves (shipworms).** *PloS one* 2009, **4:**e6085.

20. Lykidis A, Mavromatis K, Ivanova N, Anderson I, Land M, DiBartolo G, Martinez M, Lapidus A, Lucas S, Copeland A, et al: **Genome sequence and analysis of the soil cellulolytic actinomycete Thermobifida fusca YX.** *Journal of bacteriology* 2007, **189:**2477-2486.

21. Chertkov O, Sikorski J, Nolan M, Lapidus A, Lucas S, Del Rio TG, Tice H, Cheng J-F, Goodwin L, Pitluck S, et al: **Complete genome sequence of Thermomonospora curvata type strain (B9).** *Standards in genomic sciences* 2011, **4:**13-22.

22. Foster B, Pukall Ru, Abt B, Nolan M, Glavina Del Rio T, Chen F, Lucas S, Tice H, Pitluck S, Cheng J-F, et al: **Complete genome sequence of Xylanimonas cellulosilytica type strain (XIL07).** *Standards in genomic sciences* 2010, **2:**1-8.

23. Azuma Y, Hosoyama A, Matsutani M, Furuya N, Horikawa H, Harada T, Hirakawa H, Kuhara S, Matsushita K, Fujita N, Shirai M: **Whole-genome analyses reveal genetic instability of Acetobacter pasteurianus.** *Nucleic Acids Res* 2009, **37:**5768-5783.

24. Clum A, Nolan M, Lang E, Glavina Del Rio T, Tice H, Copeland A, Cheng J-F, Lucas S, Chen F, Bruce D, et al: **Complete genome sequence of Acidimicrobium ferrooxidans type strain (ICP).** *Standards in genomic sciences* 2009, **1:**38-45.

25. Valdés J, Pedroso I, Quatrini R, Dodson RJ, Tettelin H, Blake R, Eisen Ja, Holmes DS: **Acidithiobacillus ferrooxidans metabolism: from genome sequence to industrial applications.** *BMC genomics* 2008, **9:**597.

26. Land M, Lapidus A, Mayilraj S, Chen F, Copeland A, Del Rio TG, Nolan M, Lucas S, Tice H, Cheng J-F, et al: **Complete genome sequence of Actinosynnema mirum type strain (101).** *Standards in genomic sciences* 2009, **1:**46-53.

27. Wood DW, Setubal JC, Kaul R, Monks DE, Kitajima JP, Okura VK, Zhou Y, Chen L, Wood GE, Almeida NF, et al: **The genome of the natural genetic engineer Agrobacterium tumefaciens C58.** *Science (New York, NY)* 2001, **294:**2317-2323.

28. Schneiker S, Martins dos Santos ViaP, Bartels D, Bekel T, Brecht M, Buhrmester J, Chernikova TN, Denaro R, Ferrer M, Gertler C, et al: **Genome sequence of the ubiquitous hydrocarbon-degrading marine bacterium Alcanivorax borkumensis.** *Nature biotechnology* 2006, **24:**997-1004.

29. Hoeft SE, Blum JS, Stolz JF, Tabita FR, Witte B, King GM, Santini JM, Oremland RS: **Alkalilimnicola ehrlichii sp. nov., a novel, arsenite-oxidizing haloalkaliphilic gammaproteobacterium capable of chemoautotrophic or heterotrophic growth with nitrate or oxygen as the electron acceptor.** *International journal of systematic and evolutionary microbiology* 2007, **57:**504-512.

30. Fu H-l, Meng Y, Ordóñez E, Villadangos AF, Bhattacharjee H, Gil JA, Mateos LM, Rosen BP: **Properties of arsenite efflux permeases (Acr3) from Alkaliphilus metalliredigens and Corynebacterium glutamicum.** *The Journal of biological chemistry* 2009, **284:**19887-19895.

31. Klenk HP, Clayton Ra, Tomb JF, White O, Nelson KE, Ketchum Ka, Dodson RJ, Gwinn M, Hickey EK, Peterson JD, et al: **The complete genome sequence of the hyperthermophilic, sulphate-reducing archaeon Archaeoglobus fulgidus.** *Nature* 1997, **390:**364-370.

32. Mongodin EF, Shapir N, Daugherty SC, DeBoy RT, Emerson JB, Shvartzbeyn A, Radune D, Vamathevan J, Riggs F, Grinberg V, et al: **Secrets of soil survival revealed by the genome sequence of Arthrobacter aurescens TC1.** *PLoS genetics* 2006, **2:**e214.

33. Krause A, Ramakumar A, Bartels D, Battistoni F, Bekel T, Boch J, B\"ohm M, Friedrich F, Hurek T, Krause L, et al: **Complete genome of the mutualistic, N2-fixing grass endophyte Azoarcus sp. strain BH72.** *Nature biotechnology* 2006, **24:**1385-1391.

34. Liu C-T, Lee K-B, Wang Y-S, Peng M-H, Lee K-T, Suzuki S, Suzuki T, Oyaizu H: **Involvement of the azorhizobial chromosome partition gene (parA) in the onset of bacteroid differentiation during Sesbania rostrata stem nodule development.** *Applied and environmental microbiology* 2011, **77:**4371-4382.

35. Setubal JC, dos Santos P, Goldman BS, Ertesvag H, Espin G, Rubio LM, Valla S, Almeida NF, Balasubramanian D, Cromes L, et al: **Genome sequence of Azotobacter vinelandii, an obligate aerobe specialized to support diverse anaerobic metabolic processes.** *Journal of bacteriology* 2009, **191:**4534-4545.

36. Tamas I, Dedysh SN, Liesack W, Stott MB, Alam M, Murrell JC, Dunfield PF: **Complete genome sequence of Beijerinckia indica subsp. indica.** *Journal of bacteriology* 2010, **192:**4532-4533.

37. Schmitz-Esser S, Tischler P, Arnold R, Montanaro J, Wagner M, Rattei T, Horn M: **The genome of the amoeba symbiont "Candidatus Amoebophilus asiaticus" reveals common mechanisms for host cell interaction among amoeba-associated bacteria.** *Journal of bacteriology* 2010, **192:**1045-1057.

38. Tang K-h, Barry K, Chertkov O, Dalin E, Han CS, Hauser LJ, Honchak BM, Karbach LE, Land ML, Lapidus A, et al: **Complete genome sequence of the filamentous anoxygenic phototrophic bacterium Chloroflexus aurantiacus.** *BMC genomics* 2011, **12:**334.

39. Brazilian National Genome Project Consortium: **The complete genome sequence of Chromobacterium violaceum reveals remarkable and exploitable bacterial adaptability.** *Proceedings of the National Academy of Sciences of the United States of America* 2003, **100:**11660-11665.

40. Ma Y-F, Zhang Y, Zhang J-y, Chen D-w, Zhu Y, Zheng H, Wang S-y, Jiang C-y, Zhao G-p, Liu S-j: **The complete genome of Comamonas testosteroni reveals its genetic adaptations to changing environments.** *Applied and environmental microbiology* 2009, **75:**6812-6819.

41. Amadou C, Mangenot S, Glew M, Bontemps C, Capela D, Dossat C, Marchetti M, Servin B, Saad M, Schenowitz C, et al: **Genome sequence of the -rhizobium Cupriavidus taiwanensis and comparative genomics of rhizobia.** *Genome Research* 2008**:**1472-1483.

42. Welsh Ea, Liberton M, Stöckel J, Loh T, Elvitigala T, Wang C, Wollam A, Fulton RS, Clifton SW, Jacobs JM, et al: **The genome of Cyanothece 51142, a unicellular diazotrophic cyanobacterium important in the marine nitrogen cycle.** *Proceedings of the National Academy of Sciences of the United States of America* 2008, **105:**15094-15099.

43. Seshadri R, Adrian L, Fouts DE, Eisen Ja, Phillippy AM, Methe Ba, Ward NL, Nelson WC, Deboy RT, Khouri HM, et al: **Genome sequence of the PCE-dechlorinating bacterium Dehalococcoides ethenogenes.** *Science (New York, NY)* 2005, **307:**105-108.

44. Callaghan AV, Morris BEL, Pereira IAC, McInerney MJ, Austin RN, Groves JT, Kukor JJ, Suflita JM, Young LY, Zylstra GJ, Wawrik B: **The genome sequence of Desulfatibacillum alkenivorans AK-01: a blueprint for anaerobic alkane oxidation.** *Environmental Microbiology* 2012, **14:**101-113.

45. Shinoda Y, Ikenaga Y, Abe M, Naito K, Inatomi K, Furukawa K, Inui M, Yukawa H: **Complete Genome Sequence of the Dehalorespiring Bacterium Desulfitobacterium hafniense Y51 and Comparison with Dehalococcoides ethenogenes 195.** *Journal of bacteriology* 2006, **188:**2262-2274.

46. Spring S, Nolan M, Lapidus A, Glavina Del Rio T, Copeland A, Tice H, Cheng J-F, Lucas S, Land M, Chen F, et al: **Complete genome sequence of Desulfohalobium retbaense type strain (HR(100)).** *Standards in genomic sciences* 2010, **2:**38-48.

47. Copeland A, Spring S, Göker M, Schneider S, Lapidus A, Del Rio TG, Tice H, Cheng J-F, Chen F, Nolan M, et al: **Complete genome sequence of Desulfomicrobium baculatum type strain (X).** *Standards in genomic sciences* 2009, **1:**29-37.

48. Rabus R, Ruepp A, Frickey T, Rattei T, Fartmann B, Stark M, Bauer M, Zibat A, Lombardot T, Becker I, et al: **The genome of Desulfotalea psychrophila, a sulfate-reducing bacterium from permanently cold Arctic sediments.** *Environ Microbiol* 2004, **6:**887-902.

49. Junier P, Junier T, Podell S, Sims DR, Detter JC, Lykidis A, Han CS, Wigginton NS, Gaasterland T, Bernier-Latmani R: **The genome of the Gram-positive metal- and sulfate-reducing bacterium Desulfotomaculum reducens strain MI-1.** *Environmental Microbiology* 2010, **12:**2738-2754.

50. Byrne-Bailey KG, Weber Ka, Chair AH, Bose S, Knox T, Spanbauer TL, Chertkov O, Coates JD: **Completed genome sequence of the anaerobic iron-oxidizing bacterium Acidovorax ebreus strain TPSY.** *Journal of bacteriology* 2010, **192:**1475-1476.

51. Normand P, Lapierre P, Tisa LS, Gogarten JP, Alloisio N, Bagnarol E, Bassi CA, Berry AM, Bickhart DM, Choisne N, et al: **Genome characteristics of facultatively symbiotic Frankia sp. strains reflect host range and host plant biogeography.** *Genome Research* 2007**:**7-15.

52. Aklujkar M, Young ND, Holmes D, Chavan M, Risso C, Kiss HE, Han CS, Land ML, Lovley DR: **The genome of Geobacter bemidjiensis, exemplar for the subsurface clade of Geobacter species that predominate in Fe(III)-reducing subsurface environments.** *BMC genomics* 2010, **11:**490.

53. Brügger K, Chen L, Stark M, Zibat A, Redder P, Ruepp A, Awayez M, She Q, Garrett Ra, Klenk H-P: **The genome of Hyperthermus butylicus: a sulfur-reducing, peptide fermenting, neutrophilic Crenarchaeote growing up to 108 degrees C.** *Archaea (Vancouver, BC)* 2007, **2:**127-135.

54. Yi H, Xi Y, Liu J, Wang J, Wu J, Xu T, Chen W, Chen B, Lin M, Wang H, et al: **Sequence analysis of pKF3-70 in Klebsiella pneumoniae: probable origin from R100-like plasmid of Escherichia coli.** *PloS one* 2010, **5:**e8601.

55. Jimenez E, Langa S, Martin V, Arroyo R, Martin R, Fernandez L, Rodriguez JM: **Complete genome sequence of Lactobacillus fermentum CECT 5716, a probiotic strain isolated from human milk.** *Journal of bacteriology* 2010, **192:**4800.

56. Schübbe S, Williams TJ, Xie G, Kiss HE, Brettin TS, Martinez D, Ross Ca, Sch\"uler D, Cox BL, Nealson KH, Bazylinski Da: **Complete genome sequence of the chemolithoautotrophic marine magnetotactic coccus strain MC-1.** *Applied and environmental microbiology* 2009, **75:**4835-4852.

57. Singer E, Webb EA, Nelson WC, Heidelberg JF, Ivanova N, Pati A, Edwards KJ: **Genomic potential of Marinobacter aquaeolei, a biogeochemical "opportunitroph".** *Applied and environmental microbiology* 2011, **77:**2763-2771.

58. Kaneko T, Nakamura Y, Sato S, Asamizu E, Kato T, Sasamoto S, Watanabe a, Idesawa K, Ishikawa a, Kawashima K, et al: **Complete genome structure of the nitrogen-fixing symbiotic bacterium Mesorhizobium loti (supplement).** *DNA research : an international journal for rapid publication of reports on genes and genomes* 2000, **7:**381-406.

59. Auernik KS, Maezato Y, Blum PH, Kelly RM: **The genome sequence of the metal-mobilizing, extremely thermoacidophilic archaeon Metallosphaera sedula provides insights into bioleaching-associated metabolism.** *Applied and environmental microbiology* 2008, **74:**682-692.

60. Hansen EE, Lozupone Ca, Rey FE, Wu M, Guruge JL, Narra A, Goodfellow J, Zaneveld JR, McDonald DT, Goodrich Ja, et al: **Pan-genome of the dominant human gut-associated archaeon, Methanobrevibacter smithii, studied in twins.** *Proceedings of the National Academy of Sciences of the United States of America* 2011, **108 Suppl** 4599-4606.

61. Galperin MY, Cochrane GR: **Nucleic Acids Research annual Database Issue and the NAR online Molecular Biology Database Collection in 2009.** *Nucleic acids research* 2009, **37:**D1-4.

62. Saunders NFW, Thomas T, Curmi PMG, Mattick JS, Kuczek E, Slade R, Davis J, Franzmann PD, Boone D, Rusterholtz K, et al: **Mechanisms of thermal adaptation revealed from the genomes of the Antarctic Archaea Methanogenium frigidum and Methanococcoides burtonii.** *Genome Research* 2003, **13:**1580-1588.

63. Anderson I, Ulrich LE, Lupa B, Susanti D, Porat I, Hooper SD, Lykidis A, Sieprawska-Lupa M, Dharmarajan L, Goltsman E, et al: **Genomic characterization of methanomicrobiales reveals three classes of methanogens.** *PloS one* 2009, **4:**e5797.

64. Slesarev AI, Mezhevaya KV, Makarova KS, Polushin NN, Shcherbinina OV, Shakhova VV, Belova GI, Aravind L, Natale Da, Rogozin IB, et al: **The complete genome of hyperthermophile Methanopyrus kandleri AV19 and monophyly of archaeal methanogens.** *Proceedings of the National Academy of Sciences of the United States of America* 2002, **99:**4644-4649.

65. Galagan JE, Nusbaum C, Roy A, Endrizzi MG, Macdonald P, FitzHugh W, Calvo S, Engels R, Smirnov S, Atnoor D, et al: **The genome of M. acetivorans reveals extensive metabolic and physiological diversity.** *Genome Research* 2002, **12:**532-542.

66. Fricke WF, Seedorf H, Henne A, Kruer M, Liesegang H, Hedderich R, Gottschalk G, Thauer RK: **The genome sequence of Methanosphaera stadtmanae reveals why this human intestinal archaeon is restricted to methanol and H2 for methane formation and ATP synthesis.** *Journal of bacteriology* 2006, **188:**642-658.

67. Kane SR, Chakicherla AY, Chain PSG, Schmidt R, Shin MW, Legler TC, Scow KM, Larimer FW, Lucas SM, Richardson PM, Hristova KR: **Whole-genome analysis of the methyl tert-butyl ether-degrading beta-proteobacterium Methylibium petroleiphilum PM1.** *Journal of bacteriology* 2007, **189:**1931-1945.

68. Chen Y, Crombie A, Rahman MT, Dedysh SN, Liesack W, Stott MB, Alam M, Theisen AR, Murrell JC, Dunfield PF: **Complete genome sequence of the aerobic facultative methanotroph Methylocella silvestris BL2.** *Journal of bacteriology* 2010, **192:**3840-3841.

69. Campbell BJ, Smith JL, Hanson TE, Klotz MG, Stein LY, Lee CK, Wu D, Robinson JM, Khouri HM, Eisen JA, Cary SC: **Adaptations to submarine hydrothermal environments exemplified by the genome of Nautilia profundicola.** *PLoS genetics* 2009, **5:**e1000362.

70. Starkenburg SR, Larimer FW, Stein LY, Klotz MG, Chain PSG, Sayavedra-Soto La, Poret-Peterson AT, Gentry ME, Arp DJ, Ward B, Bottomley PJ: **Complete genome sequence of Nitrobacter hamburgensis X14 and comparative genomic analysis of species within the genus Nitrobacter.** *Applied and environmental microbiology* 2008, **74:**2852-2863.

71. Klotz MG, Arp DJ, Chain PS, El-Sheikh AF, Hauser LJ, Hommes NG, Larimer FW, Malfatti SA, Norton JM, Poret-Peterson AT, et al: **Complete genome sequence of the marine, chemolithoautotrophic, ammonia-oxidizing bacterium Nitrosococcus oceani ATCC 19707.** *Applied and environmental microbiology* 2006, **72:**6299-6315.

72. Chain P, Lamerdin J, Larimer F, Regala W, Lao V, Land M, Hauser L, Hooper A, Klotz M, Norton J, et al: **Complete genome sequence of the ammonia-oxidizing bacterium and obligate chemolithoautotroph Nitrosomonas europaea.** *Journal of bacteriology* 2003, **185:**2759-2773.

73. Walker CB, de la Torre JR, Klotz MG, Urakawa H, Pinel N, Arp DJ, Brochier-Armanet C, Chain PSG, Chan PP, Gollabgir a, et al: **Nitrosopumilus maritimus genome reveals unique mechanisms for nitrification and autotrophy in globally distributed marine crenarchaea.** *Proceedings of the National Academy of Sciences of the United States of America* 2010, **107:**8818-8823.

74. Norton JM, Klotz MG, Stein LY, Arp DJ, Bottomley PJ, Chain PSG, Hauser LJ, Land ML, Larimer FW, Shin MW, Starkenburg SR: **Complete genome sequence of Nitrosospira multiformis, an ammonia-oxidizing bacterium from the soil environment.** *Applied and environmental microbiology* 2008, **74:**3559-3572.

75. Meeks JC, Elhai J, Thiel T, Potts M, Larimer F, Lamerdin J, Predki P, Atlas R: **An overview of the genome of Nostoc punctiforme, a multicellular, symbiotic cyanobacterium.** *Photosynthesis Research* 2001**:**85-106.

76. Siddavattam D, Karegoudar TB, Mudde SK, Kumar N, Baddam R, Avasthi TS, Ahmed N: **Genome of a novel isolate of Paracoccus denitrificans capable of degrading N,N-dimethylformamide.** *Journal of bacteriology* 2011, **193:**5598-5599.

77. Schleheck D, Knepper TP, Eichhorn P, Cook AM: **Parvibaculum lavamentivorans DS-1T degrades centrally substituted congeners of commercial linear alkylbenzenesulfonate to sulfophenyl carboxylates and sulfophenyl dicarboxylates.** *Applied and environmental microbiology* 2007, **73:**4725-4732.

78. Kosaka T, Kato S, Shimoyama T, Ishii S, Abe T, Watanabe K: **The genome of Pelotomaculum thermopropionicum reveals niche-associated evolution in anaerobic microbiota.** *Genome Res* 2008, **18:**442-448.

79. Reysenbach A-L, Hamamura N, Podar M, Griffiths E, Ferreira S, Hochstein R, Heidelberg J, Johnson J, Mead D, Pohorille a, et al: **Complete and draft genome sequences of six members of the Aquificales.** *Journal of bacteriology* 2009, **191:**1992-1993.

80. Yagi JM, Sims D, Brettin T, Bruce D, Madsen EL: **The genome of Polaromonas naphthalenivorans strain CJ2, isolated from coal tar-contaminated sediment, reveals physiological and metabolic versatility and evolution through extensive horizontal gene transfer.** *Environmental Microbiology* 2009, **11:**2253-2270.

81. Guo W, Wang Y, Song C, Yang C, Li Q, Li B, Su W, Sun X, Song D, Yang X, Wang S: **Complete genome of Pseudomonas mendocina NK-01, which synthesizes medium-chain-length polyhydroxyalkanoates and alginate oligosaccharides.** *Journal of bacteriology* 2011, **193:**3413-3414.

82. Fitz-Gibbon ST, Ladner H, Kim U-J, Stetter KO, Simon MI, Miller JH: **Genome sequence of the hyperthermophilic crenarchaeon Pyrobaculum aerophilum.** *Proceedings of the National Academy of Sciences of the United States of America* 2002, **99:**984-989.

83. Cohen GN, Barbe Ve, Flament D, Galperin M, Heilig R, Lecompte O, Poch O, Prieur D, Qu\'erellou Je, Ripp R, et al: **An integrated analysis of the genome of the hyperthermophilic archaeon Pyrococcus abyssi.** *Molecular microbiology* 2003, **47:**1495-1512.

84. Fauvart M, Sánchez-Rodríguez A, Beullens S, Marchal K, Michiels J: **Genome Sequence of Rhizobium etli CNPAF512, a Nitrogen-Fixing Symbiont Isolated from Bean Root Nodules in Brazil.** *Journal of bacteriology* 2011, **193:**3158-3159.

85. Porter SL, Wilkinson Da, Byles ED, Wadhams GH, Taylor S, Saunders NJ, Armitage JP: **Genome sequence of Rhodobacter sphaeroides Strain WS8N.** *Journal of bacteriology* 2011, **193:**4027-4028.

86. Takeda H, Shimodaira J, Yukawa K, Hara N, Kasai D, Miyauchi K, Masai E, Fukuda M: **Dual two-component regulatory systems are involved in aromatic compound degradation in a polychlorinated-biphenyl degrader, Rhodococcus jostii RHA1.** *Journal of bacteriology* 2010, **192:**4741-4751.

87. Risso C, Sun J, Zhuang K, Mahadevan R, DeBoy R, Ismail W, Shrivastava S, Huot H, Kothari S, Daugherty S, et al: **Genome-scale comparison and constraint-based metabolic reconstruction of the facultative anaerobic Fe(III)-reducer Rhodoferax ferrireducens.** *BMC genomics* 2009, **10:**447.

88. Munk aC, Copeland A, Lucas S, Lapidus A, Del Rio TG, Barry K, Detter JC, Hammon N, Israni S, Pitluck S, et al: **Complete genome sequence of Rhodospirillum rubrum type strain (S1).** *Standards in genomic sciences* 2011, **4:**293-302.

89. Reeve W, Chain P, Ardley J, Nandesena K, Tiwari R, Malfatti S, Kiss H, Lapidus A, Co- A, Nolan M, et al: **Complete genome sequence of the Medicago microsymbiont Ensifer (Sinorhizobium) medicae strain WSM419.** *Standards in genomic sciences* 2010**:**77-86.

90. Pukall R, Lapidus A, Nolan M, Copeland A, Glavina Del Rio T, Lucas S, Chen F, Tice H, Cheng J-F, Chertkov O, et al: **Complete genome sequence of Slackia heliotrinireducens type strain (RHS 1).** *Standards in genomic sciences* 2009, **1:**234-241.

91. Sun Z, Chen X, Wang J, Zhao W, Shao Y, Wu L, Zhou Z, Sun T, Wang L, Meng H, et al: **Complete genome sequence of Streptococcus thermophilus strain ND03.** *Journal of bacteriology* 2011, **193:**793-794.

92. Chen L, Brugger K, Skovgaard M, Redder P, She Q, Torarinsson E, Greve B, Awayez M, Zibat A, Klenk HP, Garrett RA: **The genome of Sulfolobus acidocaldarius, a model organism of the Crenarchaeota.** *Journal of bacteriology* 2005, **187:**4992-4999.

93. Sikorski J, Lapidus A, Copeland A, Glavina T, Rio D, Nolan M, Lucas S, Chen F, Tice H, Cheng J-f, et al: **Complete genome sequence of Sulfurospirillum deleyianum type strain (5175T).** *Standards in genomic sciences* 2010**:**149-157.

94. Holtman CK, Chen Y, Sandoval P, Gonzales A, Nalty MS, Thomas TL, Youderian P, Golden SS: **High-Throughput Functional Analysis of the Synechococcus elongatus PCC 7942 Genome.** *DNA research* 2005, **12:**103-115.

95. Jenkins BD, Zehr JP, Gibson A, Campbell L: **Cyanobacterial assimilatory nitrate reductase gene diversity in coastal and oligotrophic marine environments.** *Environmental Microbiology* 2006, **8:**2083-2095.

96. Sieber JR, Sims DR, Han C, Kim E, Lykidis A, Lapidus AL, McDonnald E, Rohlin L, Culley DE, Gunsalus R, McInerney MJ: **The genome of Syntrophomonas wolfei: new insights into syntrophic metabolism and biohydrogen production.** *Environmental Microbiology* 2010, **12:**2289-2301.

97. McInerney MJ, Rohlin L, Mouttaki H, Kim U, Krupp RS, Rios-Hernandez L, Sieber J, Struchtemeyer CG, Bhattacharyya A, Campbell JW, Gunsalus RP: **The genome of Syntrophus aciditrophicus: life at the thermodynamic limit of microbial growth.** *Proceedings of the National Academy of Sciences of the United States of America* 2007, **104:**7600-7605.

98. Zhaxybayeva O, Swithers KS, Lapierre P, Fournier GP, Bickhart DM, DeBoy RT, Nelson KE, Nesbo CL, Doolittle WF, Gogarten JP, Noll KM: **On the chimeric nature, thermophilic origin, and phylogenetic placement of the Thermotogales.** *Proceedings of the National Academy of Sciences of the United States of America* 2009, **106:**5865-5870.

99. Muyzer G, Sorokin DY, Mavromatis K, Lapidus A, Clum A, Ivanova N, Pati A, D'Haeseleer P, Woyke T, Kyrpides NC: **Complete genome sequence of "Thioalkalivibrio sulfidophilus" HL-EbGr7.** *Standards in genomic sciences* 2011, **4:**23-35.

100. Beller HR, Chain PSG, Letain TE, Chakicherla A, Larimer FW, Richardson PM, Coleman MA, Wood AP, Kelly DP: **The Genome Sequence of the Obligately Chemolithoautotrophic, Facultatively Anaerobic Bacterium Thiobacillus denitrificans.** *Journal of bacteriology* 2006, **188:**1473-1488.

101. Scott KM, Sievert SM, Abril FN, Ball La, Barrett CJ, Blake Ra, Boller AJ, Chain PSG, Clark Ja, Davis CR, et al: **The genome of deep-sea vent chemolithoautotroph Thiomicrospira crunogena XCL-2.** *PLoS biology* 2006, **4:**e383.

102. Sievert SM, Scott KM, Klotz MG, Chain PSG, Hauser LJ, Hemp J, H\"ugler M, Land M, Lapidus A, Larimer FW, et al: **Genome of the epsilonproteobacterial chemolithoautotroph Sulfurimonas denitrificans.** *Applied and environmental microbiology* 2008, **74:**1145-1156.

103. Pappas KM, Kouvelis VN, Saunders E, Brettin TS, Bruce D, Detter C, Balakireva M, Han CS, Savvakis G, Kyrpides NC, Typas Ma: **Genome sequence of the ethanol-producing Zymomonas mobilis subsp. mobilis lectotype strain ATCC 10988.** *Journal of bacteriology* 2011, **193:**5051-5052.
