# Supplementary material for: De novo prediction of the genomic components and capabilities for microbial plant biomass degradation from (meta-)genomes
Source: Biotechnol Biofuels. 2013 Feb 15;6:24. doi: 10.1186/1754-6834-6-24 (PMC3585893; doi:10.1186/1754-6834-6-24)
Supplement: Additional file 2: Table S2 — Evaluation and meta-parameter settings of the ensembles of classifiers. The ensembles were used for feature selection and phenotype classification of the (draft) genomes and metagenomes. The macro-accuracy for each model for a discrete set of values for the parameter C was calculated in cross-validation experiments. The five best models were selected based on macro-accuracy. The mean of the exponentially transformed parameter C and the mean macro-accuracy for these five models are shown for all trained classifiers. For details on the different ensemble classifiers, see the Results section in the manuscript. [file 1754-6834-6-24-S2.doc]

**Supplementary Table S2. Evaluation and meta-parameter settings of the ensembles of classifiers used for feature selection and phenotype classification of (draft) genomes.** The macro-accuracy for each model for a discrete set of values for the parameter *C* was calculated in cross-validation experiments. The five best models were selected based on macro-accuracy. The mean exponentially transformed parameter *C* and the mean macro-accuracy for these five models are shown for all trained classifiers. For details on the different ensemble classifiers, see the result section in the manuscript.

|  | Mean parameter *C* | Mean macro-Accuracy |
| --- | --- | --- |
| eSVM_bPFAM_ | 10^-1.7^ | 0.93 |
| eSVM_fPFAM_ | 10^-1.5^ | 0.87 |
| eSVMCAZY_A | 10^-1.0^ | 0.95 |
| eSVMCAZY_B | 10^-1.9^ | 0.95 |
| eSVMCAZY_C | 10^-1.9^ | 0.94 |
| eSVMCAZY_a | 10^-1.1^ | 0.93 |
| eSVMCAZY_b | 10^-1.6^ | 0.94 |
| eSVMCAZY_c | 10^-1.8^ | 0.92 |
